# Perceived benefits and challenges of the use of immersive virtual reality Tai Chi by older Chinese and Korean immigrants

**DOI:** 10.1080/17482631.2026.2732302

**Published:** 2026-10-28

**Authors:** Yongseop Kim, Junhyoung Kim

**Affiliations:** a Department of Occupational and Recreational Therapies, University of Utah, Salt Lake City, UT, USA; b Department of Health Behavior, Texas A&M University, College Station, TX, USA

**Keywords:** Older Asian immigrant, virtual reality, Tai Chi, health benefits, qualitative study

## Abstract

**Background:**

Older Asian immigrants encounter challenges that limit access to healthcare services and experience physical inactivity that can exacerbate psychological distress. Virtual reality offers a promising technology for delivering mindfulness and physical activity interventions that promote body awareness and physical fitness.

**Objective:**

The goal of this study was to explore the perceived benefits, challenges, and cultural relevance of IVTC program use by older Chinese and Korean immigrants.

**Methods:**

Semi-structured interviews were conducted with 19 participants following their participation in a four-week IVTC intervention. Data were analyzed using comparative thematic analysis, and data trustworthiness was ensured through member checking and reflexive processes.

**Results:**

Reported health benefits were grouped into four main subthemes: (1) enhanced physical functioning and balance, (2) cognitive stimulation, (3) mental and emotional wellbeing, and (4) convenience accessibility. Reported challenges were grouped into three subthemes, including: (1) balance and coordination difficulties, (2) physical discomfort from headsets, and (3) the need for extended VR technology use instruction. Participants also discussed the cultural relevance of Tai Chi and their openness to integrating VR into their daily routine.

**Conclusion:**

The findings of this study highlight the potential feasibility and acceptability of IVTC use for enhancing the health and engagement of older Asian immigrants.

## Introduction

Older Asian immigrants in the United States often face significant psychological challenges such as depression, social isolation, and acculturative stress that are related to underutilisation of healthcare services and programmes, and adaptation challenges such as language barriers, discrimination, and a sense of disconnection from their communities (Chao et al., 2020; Clough et al., 2013; Jang et al., 2022). These challenges contribute to diminished quality of life and life satisfaction (Jung et al., 2022; Xu et al., 2023). Asian immigrant older adults have disproportionately higher rates of chronic conditions including hypertension and diabetes, with prevalence rates 1.2 to 1.7 times higher among older foreign-born Asian adults than among U.S. born older adults (Lee et al., 2020). Research has provided evidence that physical activity reduces the depression and anxiety linked to immigration stress. In particular, group-based culturally relevant physical activities foster social engagement that helps mitigate isolation (Lai et al., 2020; Liu et al., 2023). Although participation in physical activities has been proven effective in increasing the physical, social, and mental health of older adults, (Pearce et al., 2022; Ramsey et al., 2021), physical inactivity is common among older Asian immigrants due to factors such as limited leisure resources, unfamiliarity with exercise routines, and cultural perceptions of physical activity (Maricar et al., 2024). To address these challenges, culturally relevant, accessible, and engaging forms of physical activity must be made available to this population.

One of the physical activities that has been found to be effective in increasing the health and wellbeing of older Asian immigrants is Tai Chi, a traditional Chinese mind-body exercise. Tai Chi involves a series of choreographed movements with gentle weight shifting, precise body alignment, controlled transition, and mindful attention to breath and posture. Substantial research evidence indicates that the focus of Tai Chi on mindfulness, strength and balance, structural alignment, and flexibility is well aligned with the needs of geriatric populations (Bai et al., 2023; Qu et al., 2024; Salmoirago-Blotcher et al., 2021). Multiple empirical studies have provided evidence for the effectiveness of Tai Chi in improving mindfulness and body awareness and enhancing physical fitness including lower body balance, strength, and flexibility (Bai et al., 2023; Chewning et al., 2020). For example, a group of older adults who regularly engaged in Tai Chi exercise an average of 25 minutes a day, six days a week reported significantly improved leg strength, tandem balance, mobility, gait, balance confidence, and executive functioning (Chewning et al., 2020).

Despite the health benefits of Tai Chi participation, there are structural, programmatic, and environmental barriers that can impede participation in Tai Chi by older Asian immigrants. Barriers to participation may include a lack of individualisation, transportation to exercise locations, and the limited availability of instructors or structured programmes in culturally accessible settings (Cerin et al., 2019; Horne & Tierney, 2012; Zou et al., 2021). To minimise these barriers, virtual reality (VR) technology has emerged as a promising technology for the facilitation of higher levels of exercise participation by older adults as VR-based applications offer customisable, immersive environments that can increase motivation and promote engagement. For example, older adults can engage in guided or immersive VR activities that promote psychological wellbeing without needing to travel to community centres or healthcare facilities (Chitjamnogchai et al., 2025; Gao et al., 2024). Multiple studies have provided empirical evidence that immersive VR exercise programmes significantly improve attributes such as balance, muscle strength, and overall physical performance (Agbangla et al., 2022; Campo-Prieto et al., 2022; Sadeghi et al., 2021). This is particularly relevant for older adults who encounter barriers to engaging in physical activities that are related to mobility challenges or a lack of motivation. The interactive nature of VR provides a stimulating environment that can lead to increased enjoyment of and engagement in exercise that is of crucial importance to this population (Campo-Prieto et al., 2022).

The findings of a recent pilot study that was conducted to investigate the health benefits of participation in an immersive VR Tai Chi programme by older adults during the COVID-19 pandemic suggest that Tai Chi can be instrumental in improving mindfulness and enjoyment in physical activity (Wang et al., 2024). This pilot study provides evidence that an immersive VR Tai Chi programme can contribute to the physical and mental health of older adults. While most VR and VR Tai Chi studies provided evidence of the efficacy of VR programmes in providing health benefits, few studies have adequately addressed the cultural barriers that shape the participation of older Asian immigrants. It is important to expand the availability of the benefits that can be derived from participation in IVTC by older Asian immigrants and contribute to the design and implementation of VR-based programmes that address the health disparities experienced by older Asian immigrants. For this study, we selected older Chinese and Korean immigrants as, according to the U.S. Census Bureau (2020), the number of older Asian immigrants in the United States has reached a record high of 2.4 million, which makes them one of the fastest-growing aging populations in the U.S. Despite this growth, older Chinese and Korean adults face persistent disparities in health outcomes compared to their non-immigrant counterparts that are largely related to limited access to community-based recreational programmes and culturally adapted healthcare services.

Therefore, the purpose of this study was to qualitatively investigate the health benefits and challenges associated with IVTC participation by older Chinese and Korean immigrants. Through our exploration of these health benefits and challenges, we provide suggestive evidence of how participation in an IVTC programme can be instrumental in improving health and how this programme can be modified, developed, and refined. In addition, the findings of this study provide critically needed empirical evidence that will provide the foundation for the design and testing of the efficacy of the programme in a large-scale clinical trial.

## Methodology

### Study design

We employed in-depth interviews to explore the health benefits and challenges experienced by community-dwelling older Chinese and Korean immigrant adults. The interview approach and protocol were informed by prior qualitative studies examining the use of technology-based Tai Chi interventions by older Korean immigrants (Kim & Kim, 2021; Kim et al., 2015, 2019) that demonstrated the utility of in-depth interviews for capturing culturally grounded health perceptions and experiential outcomes. Building on this earlier work, we extended the methodology to a broader immigrant population and a virtual reality-based intervention context.

### Participants

A total of 24 participants were initially recruited (22 Chinese, 2 Korean; 7 male, 17 female) with a mean age of 73, a maximum age of 84, and a minimum of 65. Of the 24 participants recruited, 19 completed the post-intervention interviews. Five participants did not complete the interviews due to scheduling conflicts, health issues, or withdrawal from the programme. Participants were recruited through a community-based Senior Recreation Community Centre in Texas that provides a variety of recreational and educational programmes for older Asian immigrants. Administrative databases were used to identify participants over 65 years of age and exclude potential participants who were living with severe psychiatric disorders. Eligibility criteria for potential participants included those who: a) were over the age of 65 and had immigrated from China or South Korea, b) had no serious chronic physical or mental health disorders, c) had no uncorrected hearing or visual impairment, d) had a Montreal Cognitive Assessment (MoCA) score above 25 (indicating no cognitive impairment), and e) had no VR experience within the past year. A total of 19 participants who completed the programme participated in the interview (see Table I). Ethical approval for this study was granted by the Institutional Review Board of Indiana University..

**Table I.** Demographic table.

Participant ID	Age	Gender	Country of Origin	Years in U.S.
P01	65	Male	China	3
P02	84	Male	China	5
P03	65	Female	China	6
P04	76	Female	China	5
P05	77	Female	China	6
P06	70	Female	China	4
P07	74	Female	Korea	1
P08	68	Male	China	8
P09	72	Female	China	2
P10	80	Female	China	1
P11	72	Female	China	4
P12	72	Female	China	5
P13	76	Male	Korea	3
P14	73	Female	Korea	3
P15	70	Female	China	2
P16	72	Female	China	4
P17	75	Female	China	10
P18	79	Male	China	2
P19	67	Female	Korea	2

### Immersive virtual reality Tai Chi intervention

Based on a previous IVTC study protocol, our team designed and implemented a four-week intervention that consisted of eight, twice weekly 30-minute sessions. The IVTC programme was developed with Yang-style Tai Chi techniques that included 35 gentle movements involving coordinated arm and leg postures that controlled lower and upper body movements. The intervention was guided by a virtual Tai Chi instructor whose movements were displayed from both front and side perspectives to support accurate learning and imitation. Participants followed recorded audio instructions during each session that was conducted in nature-inspired, immersive environments featuring over 30 different settings such as mountains, beaches, forests, and cityscapes to allow for participant preference. Background audio included ambient sounds that enhanced the calming and engaging experience. Participants chose session themes including “calm” or “energising” to suit their desired emotional state and energy level. The Tai Chi routines were structured into timed segments with each movement demonstrated at a steady, easy-to-follow pace using a Guided Tai Chi application that is commercially available on MetaQuest.com. Participants wore Meta Quest 2 VR headsets and used two handheld controllers that tracked body movements in real time. Sessions were conducted in a supervised setting, at the participant’s care facilities with research staff available on-site to provide technical support and ensure participant safety.

### Procedures

Research staff coordinated with facility administrators to introduce the IVTC programme. Flyers were posted in common areas such as the staff room and living room to allow potential participants to learn about the programme. The facility director and staff members regularly announced the programme during morning sessions, and interested individuals were signed up, screened for eligibility, and provided their written informed consent prior to participation.

Before the first session, all participants were provided with informed consent documents that were translated into Chinese and Korean to ensure full comprehension. The forms explicitly stated that their agreement was required for participant data to be used in published research. Participants were given time to review the documents and sign them prior to beginning the first session. All signed consent forms were collected by the research staff before the intervention began. Consent to publish anonymized data and quotations was obtained from each participant individually, and all identifiable information was removed to protect participant confidentiality.

The research staff conducted an orientation session to provide information about the VR devices and the IVTC programme. Participants received visualised instruction guides, live demonstrations, and hands-on practice using the Meta Quest 2 headsets and controllers. Screen casting was employed to allow the research staff to observe what participants were viewing and doing in the VR environment in real time. Participants were guided through each step of device use including wearing the headset, holding the controllers, and navigating the virtual menu. Each device was set up on an assigned table and chair, with a clearly marked area of 6.5 feet by 6.5 feet to ensure safe movement during Tai Chi practice.(see Figure 1). During each session, research staff were present to assist with programme and device setup, monitor safety, and address any technical issues. Attendance was recorded, and participants were encouraged to provide feedback after each session.

**Figure 1.**
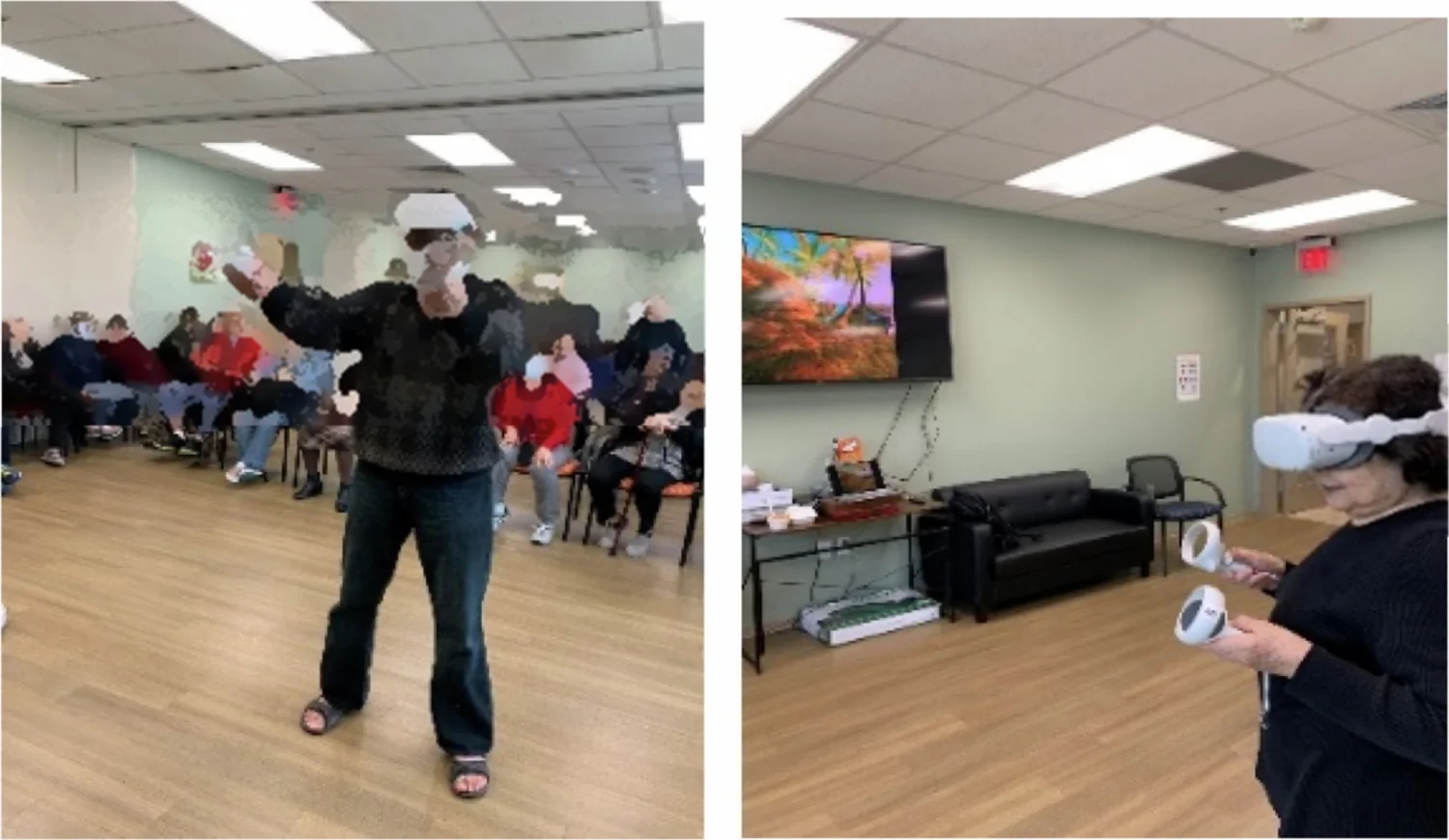
IVTC participation.

### Data collection

Of the 24 recruited participants, 19 completed the programme and were interviewed using semi-structured interviews to gain in-depth information about their experiences with the IVTC programme. 30-to-60-minute interviews were designed to elicit detailed feedback on participant experiences with the IVTC programme that included perceived benefits, motivational aspects, usability challenges, and overall satisfaction. Each interview was audio-recorded with consent, transcribed verbatim, and analysed using qualitative methods. The interview guide was developed by the research team in collaboration with experts in technology-based interventions for geriatric populations.

The interview protocol included both content mapping and content mining interview questions to capture a comprehensive understanding of participant experiences. Content mapping questions were used at the beginning of each interview to establish a broad overview of participant experiences and to identify key topical domains. Examples of questions included: “What were your overall experiences using VR for Tai Chi? Could you elaborate on your experience?” “What potential benefits did you gain from participating in the IVTC programme?” and “What challenges did you encounter when participating in IVTC?”. These questions allowed the research teams to establish a general overview of participant experiences and their perceptions of VR Tai Chi. Content mining involves the use of in-depth probing questions to explore specific areas of experience in greater detail. Once the main themes were mapped, content mining uncovered mechanisms and nuances. This process was conducted by the interviewer through follow-up probes and clarification questions tailored to the responses of each participant. Examples of content minining questions included, “Can you describe how this activity affected your physical comfort and balance?”, “What aspects of the programme made it more or less motivating for you?” (Legard et al., 2003). After each interview, the participants completed brief demographic questionnaires that captured information including age, sex, and educational background. Interview questions were translated from English into the native language of the study participants (either Chinese or Korean) by the interviewer and responses were then translated back into English by the interviewer after completion of the interview.

### Data analysis

Interview data were analysed using a thematic approach guided by the constant comparative analysis techniques proposed by Cresswell (2013). The team used a constant comparative method to refine themes and ensure consistency across transcripts that included: (a) preparing raw data for analysis, (b) understanding and analysing each data item, (c) generating general themes with direct quotes, and (d) interpreting the themes. Transcripts were independently reviewed and coded by multiple researchers. Preliminary codes were developed and then redefined into broader themes and subthemes through iterative team discussion. To enhance data analysis trustworthiness and credibility, member-checking and peer debriefing were used as suggested by Birt et al. (2016). In addition, our research team was provided with rigorous training and had extensive experience using qualitative methods.

## Findings

Our thematic analysis produced seven subthemes describing how participants benefited and experienced challenges associated with their IVTC participation. As regards health benefits, three subthemes were identified: (1) enhanced physical functioning and balance, (2) cognitive stimulation, (3) mental and emotional wellbeing. In addition, convenient accessibility to testing was also identified as a key benefit. These benefits indicate that the IVTC programme is instrumental in improving the health and wellbeing of older Chinese and Korean users. In addition, we captured several challenges experienced by participants including: (1) balance and coordination difficulties, (2) physical discomfort from headsets, and (3) the need for extended instruction. These findings provide a foundation for refining the programme protocol and enhancing its feasibility and usability in future clinical trials (see Table II).

**Table II.** Themes, subthemes, and descriptions of participants’ perceived benefits and challenges of virtual reality Tai Chi.

Theme	Subtheme	Description
Benefits	Enhanced physical functioning and balance	Improvements in posture, stability, and confidence in movement.
	Cognitive stimulation	Learning and using VR technology to stay mentally active
	Mental and emotional wellbeing	Reduced stress, improved mood, and emotional relief from immersive practice.
	Convenience accessibility	Ability to choose preferred backgrounds and music to match mood or energy.
Challenges	Balance and coordination difficulties	Difficulty synchronising Tai Chi movements with VR cues, especially for those with mobility issues.
	Physical discomfort from headsets	Heaviness or eye fatigue resulting from prolonged use.
	Need for extended instruction on VR technology use	Desire for more training and consistent guidance in using the VR equipment.

### Enhanced physical functioning and balance

All participants reported a variety of health benefits such as improved physical movements and posture stability that were related to IVTC programme participation. They mentioned that they enjoyed engaging in physical movement by following virtual Tai Chi instructions and believed that this programme helped them become more physically active and improved their balance. They stated that the gentle body movements characteristic of Yang-style Tai Chi helped them engage in slow and deliberate motions and felt that they had developed improved balance skills. Several participants believed that the programme was beneficial for their physical functioning due to maintaining a steady posture and improvements in physical stability. One participant said,

*Because the practice focused on slow steps, I felt I had more control over my body. Even on uneven ground or stairs, I could balance myself better than before after doing the [IVTC] programme.*

This suggests that IVTC may support proprioceptive awareness and balance confidence rather than merely providing physical exercises.

While some participants noted that the movements were not as effective or identical to those in traditional Tai Chi, others reported that using the controllers offered movements and the benefits comparable to traditional Tai Chi. For example, one participant mentioned,

*[Using controllers] definitely [helpful], because if my feet can’t keep up, I can use my hands to help balance.*

Interestingly, the variation of participants’ perceptions of authenticity of Tai Chi movements reflect a broader negotiation between traditional embodied practice and technologically mediated adaptation. This suggest that perceived effectiveness may be influenced by their previous Tai Chi experience and expectations of authenticity.

### Cognitive stimulation

Several participants mentioned that they enjoyed engaging in immersive environments that stimulated their ability to concentrate on Tai Chi movements and that the provided authentic landscapes surrounding them allowed them to focus on programme activities. In addition, they reported that trying something new allowed them to experience a new kind of stimulation as a brain exercise. One participant shared,

*It stimulates your brain and lets you learn something new, which is good for older people. It can help spark interest, so that’s a positive.*

These statements suggest that novelty itself may operate as a cognitive stimulus. The act of adapting to new visual-spatial environments, synchronising movement with visual cues, and navigating immersive space may have contributed to perceived mental engagement.

Some participants highlighted that focusing on the Tai Chi movements on the screen helped them to remain aware of their surroundings. However, they also noted that the pace of the programme sometimes felt too slow.

*It helps me focus and concentrate on the Tai Chi movement, but sometimes the waiting time before the next step feels too long. I think having other activities or more engaging movements would help me stay interested and keep my eyes focused. I think that might also be more interesting to others.*

This tension indicates that cognitive stimulation is not universally experienced; it may depend on alignment between programme tempo and individual cognitive expectations. Thus, stimulation appears to be mediated by perceived challenge level rather than immersion alone.

### Mental and emotional wellbeing

Most importantly, many participants believed that participating in the IVTC programme helped them to remain calm and relaxed, which contributed to their mental health and wellbeing, particularly when they chose Tai Chi practice settings such as mountains, beaches and hills, with music that allowed them to experience a sense of relaxation and mindfulness. One participant said,

*This helps me to relax because of the colours [in the background] and the movement that I follow.*

He also indicated that the calming background environments played an important role in increasing his motivation to follow the modelled physical movements.

Participants reported enjoying the unique VR immersive environments that allowed them to fully engage in Tai Chi movements and gain relaxation. For example, one participant emphasised the emotional and sensory value of the experience and stated,

*The field of vision is very wide… It’s dreamy, beautiful, and makes you feel emotionally better… You can do Tai Chi while surrounded by a mountain or watching the scenery at the beach. The scenery feels real—almost like being at the actual beach.*

These responses suggest that VR environments functioned as emotionally regulating spaces, providing simulated access to nature and expansive landscapes that may otherwise be inaccessible. The immersive visual field appears to amplify the meditative qualities of Tai Chi, potentially enhancing mindfulness-like experiences.

In a similar manner, other participants expressed that they experienced a reduction in their stress while engaging in the programme, and that the gentle movements and selected backgrounds helped relieve their stress. According to one participant,

*Tai Chi helps relieve [my] stress and improves [my] emotional health when I do the slow movements.*

emphasising the emotional benefits that she had gained from virtual VR programme participation.

While some participants had initially believed VR to be complex and unfamiliar, they realised its potential to stimulate cognitive engagement and promote overall wellbeing. Several participants valued the process of learning and interacting with new technology and considered this activity to be intellectually stimulating. They thought that engaging with the IVTC programme not only increased their ability to perform physical movements, but that it also supported brain activities due to the exploration of new VR-based settings. For example, one participant stated,

*It stimulates your brain and lets you learn something new, which is good for older people. It can help spark interest, so that is a positive.*

Some participants were able to fully immerse themselves in the programme due to the calming tempo and immersive VR environment that was a novel experience. They mentioned that the virtual Tai Chi programme was innovative and motivational for them due to its inherent novelty and the fun features included in the programme. According to a participant,

*I found the overall experience refreshing. It felt like high-tech, and I really liked it… Yes, because I had never seen anything like it before. It is very novel, isn’t it?*

These statements indicate that participants viewed the programme as a fun, engaging, and novel activity and expressed satisfaction and enjoyment with the programme, providing evidence that participants benefitted from the characteristics that are inherent to VR-based experiences.

*The advantage is that when I wear the VR headset, the 3D effect is quite strong. It feels like I’m outdoors. It gives a luxurious feeling, right? That part feels quite good.*

These examples provide suggestive evidence that engaging with the IVTC programme allowed our older Asian immigrant participants to experience a sense of relaxation and mindfulness, VR-based learning engagement, and many other potential mental health benefits from their participation in the IVTC programme.

### Convenience and accessibility

Many participants recognised the potential of the programme as a home-based exercise activity due to its convenience and accessibility. They mentioned that they were able to wear the VR gear in an effective manner and engage in the activities without difficulty. They also highlighted the advantage of consistent practice without the limitations of space, time, and financial resources. One participant remarked about the flexibility that the programme provided, especially as compared to in-person classes that require scheduling and travel.

Another participant shared,

*It is quite good! At least you do not need to find an instructor, right? You can just use the VR and practice by yourself. I attend a class at Songlin College every Thursday morning, where a master comes to teach. However, he often misses classes for personal reasons, which makes it hard to maintain consistency. The benefit of VR is that it is virtual, so in this situation, you do not have to worry about the teacher's schedule.*

Another participant appreciated the opportunity to practice the Tai Chi activity in a virtual format. She shared her idea that this virtual Tai Chi programme allowed her to select the practice setting, which minimised structural and environmental barriers. She stated,

*For people who have a small home without their own yard… seeing the vast ocean could be a good thing*.

These statements highlight the perceived convenience and accessibility of the VR Tai Chi programme that minimised the barriers that older adults can experience when participating in traditional, in-person Tai Chi classes and provides suggestive evidence that the use of a VR Tai Chi programme can be a promising alternative for expanding access to evidence-based exercise programmes. Also, IVTC may function as a structural equaliser, offering culturally relevant exercise without dependence on external resources. For immigrant older adults who may face linguistic or mobility constraints, VR delivery potentially reduces participation barriers.

### Balance and coordination difficulties

Despite the potential for the health benefits that can be realised through IVTC participation, some participants identified challenges associated with their participation such as physical discomfort and difficulty operating the VR equipment. Some participants noted that the headset display occasionally contributed to a sense of falling that led to imbalance, dizziness, or eye strain. These issues raised concerns regarding their ability to maintain balance and safely engage with the programme. For example, one participant stated,

*I feel that for us older adults, adapting to this seems a bit difficult. Why? Because in Tai Chi, both hands and feet need to follow the movements displayed in the programme. Especially my feet, as I have some issues with balance, which makes it easy for me to fall. Overall, I feel that the VR experience is a bit challenging for me.*

Some participants experienced dizziness after using the IVTC programme in the VR environment that made them feel hesitant and less likely to use it in the future, for example,

*I don’t really enjoy playing it; wearing the headset makes my head feel dizzy after a while. [I am not going to use it in the future], Because of the impact on my eyes and dizziness, I don’t think I could handle it.*

Also, another participant mentioned,

*Maybe it’s because of my age, but I felt dizzy—so much so that I felt like I was going to fall. It was as if I might topple over. For instance, I thought there was a desk in front of me. I practice Tai Chi daily, but it was displayed too slowly—way too slow.*

Additionally, some participants thought that the IVTC was not as effective as traditional exercise programmes because they perceived the pace to be too slow. However, this was attributed not to the programme itself, but to the nature of Tai Chi in general.

*It felt like there was no exercise benefit because it was slower than keeping tempo with the movements. I even wondered if Tai Chi could really count as exercise. The movements didn’t feel like they would result in effective exercise.*

These reports underscore that while IVTC may offer opportunities for physical engagement, older adults may experience sensory related barriers that may limit sustained participation.

### Physical discomfort from headset

While many participants noted that the headset adjustments didn’t cause much pressure and felt relatively comfortable, several participants noted that the heavy weight of the device and the pressure it placed on their forehead contributed to discomfort during use. Others reported experiencing dizziness or headaches that they attributed to both the tight fit and the overall weight of the headset. These factors made it challenging for them to wear the device for extended periods of time. For example, one participant reported,

*The headset was heavy, and after some time, it pressed on my forehead and made me uncomfortable.*

Also, another participant highlighted the intersection of device weight and physical instability, noting,

*The headset felt very heavy after ten minutes. I could feel the pressure right on my brow bone, and it made it hard to focus on the exercises. If [the strap] was loose, it wobbled and made me feel dizzy.*

Overall, many participants mentioned that the VR head strap supported their movements and was manageable; however, prolonged use of the headset often led to head pressure that may pose potential barriers to sustaining VR use by older adults. These findings highlight the importance of integrating considerations of comfort in the ergonomic design of VR equipment and applications to enhance long-term feasibility and sustained adoption.

### Need for extended instruction on VR technology use

A small number of participants shared that they needed longer educational sessions on how to operate the VR gear and its functions. They shared that they had been anxious about using the VR headsets due to unfamiliarity with the technology and general sense of technology anxiety that was caused by not knowing how to troubleshoot the device. For example,

*It is still uncertain how to operate it. You need to teach how to use it properly.*

The other participant also mentioned,

*I think high-tech is a challenge for our generation. It is a bit complicated, and sometimes we need help.*

Another participant experienced that it took some time to become used to using the VR gear in an appropriate manner. Even though it took some time, she said that once comfortable, she was able to fully engage in the activity. She stated,

*I tried to adjust it myself but couldn’t. I still needed their help… the biggest issue is not knowing how to use this properly or how to resolve problems when they arise.*

Despite the potential health benefits that can be accrued by users of the programme, these statements show that participants experienced some challenges to their use of the VR programme. These identified challenges need to be addressed in the further development and refinement of the programme prior to future clinical trials.

## Discussion

We have identified the benefits and challenges associated with IVTC programme participation by older Chinese and Korean immigrants. The findings of our study provide suggestive evidence that older Chinese and Korean immigrants can obtain physical, cognitive, and mental health benefits through IVTC participation and that the unique and innovative features of the IVTC programme such as selections of practice setting, sound, or music and the presence of a virtual instructor served as important sources of enjoyment, motivation, and sustained engagement. Overall, our findings suggest the potential of IVTC programme use in promoting the health and wellbeing of older Chinese and Korean users.

Recent VR-based studies have demonstrated the potential of various types of VR interventions to provide health benefits for older adults, even those with cognitive impairment (Chang et al., 2022; Kwan et al., 2024; Liao et al., 2019). While these studies have provided evidence of the efficacy of VR programmes for health, our study expands previous findings that older immigrants can gain similar health benefits with a VR-based programme.

To date, little research has been conducted to investigate the potential of VR application use to provide health benefits to older Chinese and Korean immigrants. The findings of this study suggest that many participants viewed using the VR programme as a manageable and culturally acceptable tool. In particular, features such as accessibility, guided instruction, and an immersive environment appear to have reduced the barriers commonly faced by older adult immigrants. By minimising these barriers, IVTC programme use shows promise for enhancing the physical activity levels of older adult immigrants. It is important to note that IVTC programme use can be instrumental in promoting improved physical, cognitive, and mental health in older immigrants. Our findings are aligned with a growing body of evidence demonstrating the potential of VR-based interventions to promote the physical health of older adults. Earlier studies have shown that VR exercise programmes can improve balance, mobility, and cognitive functioning, even in populations with mild cognitive impairment (Chang et al., 2022; Kwan et al., 2024; Liao et al., 2019). Similar to these findings, participants in our study reported experiencing improvements in balance, focus, and relaxation through IVTC participation. This is consistent with earlier studies showing that 2D or online Tai Chi programmes can enhance both the physical function and mental health outcomes of older adults (Li et al., 2022; Wang, 2024; Zhu et al., 2024). However, our findings add nuance by providing evidence that immersive VR features such as interactive landscapes, guided instruction, and 3D visualisations provide additional motivation and engagement beyond what traditional or 2D programmes offer. Importantly, our study extends this literature by highlighting that these benefits are also accessible to older immigrant populations who may face additional cultural and linguistic barriers to participation in community-based health programmes.

The Centres for Disease Control and Prevention (2017) suggests that traditional Tai Chi is one of the most effective balance exercises for older adults to use to reduce the risk of falls. Substantial research evidence supports this recommendation by demonstrating that Tai Chi improves physical functioning (including balance, strength, and flexibility) and enhances the mental health of older adult participants (Bai et al., 2023; Qu et al., 2024; Salmoirago-Blotcher et al., 2021). Our study provides additional evidence that participants in the IVTC programme experienced similar benefits, particularly in balance and structural alignment, which suggests that VR-delivered Tai Chi can replicate some of the core health advantages traditionally associated with in-person practice.

Chinese immigrant study participants tended to highlight the structural and traditional aspects of Tai Chi such as precision movement and alignment with cultural practices. In contrast, Korean immigrant participants more often emphasised the social and motivational aspects including accessibility of the programme, convenience of practicing at home or community settings, and the sense of encouragement provided by the instructor and virtual landscapes. Despite these nuanced differences, both groups viewed IVTC as a culturally acceptable and valuable means of engaging in physical activity.

A small number of earlier studies have focused on the ability of two-dimensional Tai Chi programmes to produce health benefits in community-dwelling older adults (Lee et al., 2024; Qu et al., 2024; Wang et al., 2024; Zhu et al., 2024). These studies provided empirical evidence of the efficacy of two-dimensional Tai Chi in improving the physical functioning and enhancing the mental health of older adult participants. Kim et al. (2022) conducted a study in which the efficacy of IVTC programme use was evaluated by Tai Chi experts and indicated that IVTC participants experienced an improved sense of enjoyment, convenience, and accessibility, and highlighted the appropriateness of such a programme for Tai Chi beginners. The findings of this study provide additional support to the contention that IVTC programme use can help older immigrants gain health benefits and unfettered access to Tai Chi.

While qualitative research does not seek statistical generalisability, our findings may have transferability to other contexts. The cultural and technological factors identified in this study could inform the design of VR-based exercise interventions for other immigrant groups or older minority adult populations who encounter similar barriers to physical activity such as language limitations, transportation challenges, or limited culturally relevant programming. The immersive and culturally customisable nature of IVTC could be adapted for different cultural traditions such as yoga for South Asian immigrants and Qigong for other East Asian groups that can be delivered in a variety of settings including community centres, senior centres, community health clinics, and home-based programmes. In addition, we noted that technology anxiety, equipment discomfort, and the need for comprehensive orientation are not unique to Chinese and Korean participants, and that addressing these challenges in programme design has the potential to enhance the accessibility of such interventions for broader populations. Other studies reported challenges with VR-based intervention use by older adults including discomfort from headset weight, difficulties with system navigation, and concerns about dizziness or disorientation (Appel et al., 2020; Huygelier et al., 2019). Addressing these barriers in programme design may enhance accessibility and engagement for broader populations. In our study, the use of Tai Chi as the intervention may have reduced cultural resistance as it represented a familiar and culturally valued practice for participants. Further, improvements in VR headset ergonomics, such as lighter-weight devices and adjustable straps, may also contribute to reducing discomfort and facilitating adaptation by older adults (Bailenson, 2018).

Despite these positive outcomes, several limitations of our study must be addressed. First, the inherent limitation of qualitative methodology as regards the generalisability of our study findings must be acknowledged. Future quantitative or mixed-methods studies are needed to determine the extent to which IVTC programme participation provides health benefits to older Asian immigrants. Second, individual factors such as acculturation levels, attitudes toward technology use, and current physical limitations and cognitive functioning status can affect the quality and efficacy of participation in an IVTC programme and its resultant health outcomes. It would be beneficial if future researchers investigated how these variables impact health outcomes that are associated with IVTC programme participation. Third, our study relied on self-reported data from semi-structured interviews that introduces the potential for social desirability bias. Because the sessions were conducted with research staff on-site to provide guidance, and technical assistance, participant reports of satisfaction may have been influenced by the presence of study personnel. Participants may have felt inclined to express positive experiences in order to appear cooperative or to please the research team. This limitation is particularly salient given the long-term goal of implementing IVTC programme in unsupervised or home-based environments. Fourth, the lack of a control group limits our ability to determine whether the observed benefits were specifically attributable to the IVTC programme or whether similar outcomes might have been achieved through other forms of activity such as traditional Tai Chi classes, social engagement, or general exercise participation. Controlled trials will be essential to establishing causal effects. Fifth, we focused on two sub-populations as a sample and identified a lack of understanding of IVTC programme use among older immigrant groups. Expanding the impact of the programme to other immigrant populations will produce a comprehensive overview of the impact of VR programme participation on health outcomes on the overall immigrant population. Lastly, translation of interview data was conducted by a single bilingual interviewer without formal back-translation or independent verification. While the interviewer was familiar with the cultural and linguistic context of participants, the lack of cross-validation procedures may have introduced potential bias or minor loss of linguistic nuance.

Future quantitative and mixed-methods studies are needed to determine the extent to which IVTC programme participation provides measurable health benefits for older Asian immigrants. In particular, it would be valuable for future researchers to investigate how individual factors such as acculturation levels, attitudes toward technology use, and physical or cognitive status affect both participation quality and the health outcomes associated with IVTC use. Moreover, testing the IVTC programme in larger and more diverse populations will be essential for evaluating its broader applicability and cultural relevance. In addition, longitudinal studies are needed to assess the sustained impact of IVTC participation on physical, cognitive, and mental health outcomes, as well as on adherence and engagement over time.

Our study provides much-needed evidence of the potential impacts and challenges associated with IVTC programme participation by older Chinese and Korean immigrants and provides invaluable insights into and practical suggestions for future programme design. Addressing the challenges we uncovered that may negatively affect participant engagement with an IVTC programme can be used to provide future programme refinements. Overall, our study provides suggestive evidence that IVTC programme participation has significant potential to improve the health and wellbeing of older Chinese and Korean immigrants and provide a foundation for refining, designing, and demonstrating the efficacy of IVTC programme use in a large-scale clinical trial.

## Data Availability

The data supporting the findings of this study are available upon request from the corresponding author, YK. These data are not publicly available as they contain identifiable participant information that could compromise the participant privacy.
